# Intravenous immunoglobulin for the acute treatment of refractory optic neuritis in Japan

**DOI:** 10.1007/s10384-025-01210-6

**Published:** 2025-05-16

**Authors:** Yohei Takahashi, Takeshi Kezuka, Keigo Shikishima, Akiko Yamagami, Hideki Chuman, Makoto Nakamura, Satoshi Ueki, Akiko Kimura, Masato Hashimoto, Sonoko Tatsui, Nobuyuki Shoji, Hitoshi Ishikawa, Collaborative Research Group: Investigators, Yohei Takahashi, Takeshi Kezuka, Keigo Shikishima, Akiko Yamagami, Hideki Chuman, Makoto Nakamura, Satoshi Ueki, Akiko Kimura, Masato Hashimoto, Hitoshi Ishikawa, Kimiyo Mashimo, Akinori Baba, Yasuyuki Iguchi, Teppei Komatsu, Mayumi Iwasa, Yuko Nakanishi, Mari Sakamoto, Sotaro Mori, Norio Chihara, Akiko Masuda, Yoshihito Mochizuki, Takeo Fukuchi, Yoichiro Shinohara, Kiyotaka Nakamagoe, Shigenari Suzuki, Norito Kokubun, Takahiko Yamanoi, Takashi Iizuka, Hirotaka Yokouchi, Ryutaro Akiba, Junichiro Kizaki, Hidetoshi Onda, Eiji Tomoyori, Yuji Inoue, Yurika Aoyama, Hiromasa Sawamura, Makoto Aihara, Tadashi Matsumoto, Toru Kurokawa, Yuichi Toriyama, Marie Nakamura, Mitsuya Otsuka, Akiko Hikoya, Miwa Komori, Hidehiro Oku, Takahisa Hirokawa, Eri Nakano, Kenji Suda, Atsushi Miki, Katsutoshi Goto, Ken Fukuda, Kayo Sugiura, Norimitsu Fujii, Yuka Sogabe, Koichiro Tamura, Naoya Imanaga

**Affiliations:** 1 Department of Ophthalmology, Kitasato University School of Medicine, 1-15-1 Kitazato, Minami-ku, Sagamihara-shi, Kanagawa, 252-0373 Japan; 2 Department of Ophthalmology, Tokyo Women’s Medical University, Tokyo, Japan; 3 Department of Ophthalmology, Tokyo Medical University, Tokyo, Japan; 4 Department of Ophthalmology, The Jikei University School of Medicine, Tokyo, Japan; 5 Inouye Eye Hospital, Tokyo, Japan; 6 Department of Ophthalmology, University of Miyazaki, Miyazaki, Japan; 7 Division of Ophthalmology, Department of Surgery, Kobe University Graduate School of Medicine, Hyogo, Japan; 8 Department of Ophthalmology, Niigata University, Niigata, Japan; 9 Department of Ophthalmology, Hyogo Medical University, Hyogo, Japan; 10 Department of Ophthalmology, Nakamura Memorial Hospital, Hokkaido, Japan; 11 Department of Orthoptics and Visual Science, Kitasato University School of Allied Health Sciences, Kanagawa, Japan

**Keywords:** Intravenous immunoglobulin, Optic neuritis, Steroid-resistant, Plasmapheresis

## Abstract

**Purpose:**

To investigate the usage status and evaluate the efficacy of intravenous immunoglobulin (IVIG) for the acute treatment of optic neuritis (ON) in Japan.

**Study design:**

Multicenter retrospective case series.

**Methods:**

The study subjects were patients with steroid-resistant acute ON in whom IVIG had been initiated between January 2020 and August 2022 at 30 facilities in Japan. The clinical characteristics, visual acuity, and adverse events following IVIG were compared among anti-aquaporin 4 antibody positive ON (AQP4-ON), anti-myelin oligodendrocyte glycoprotein antibody positive ON (MOG-ON), and idiopathic ON (ION).

**Results:**

The study included sixty-five patients (76 eyes); the main clinical department administering IVIG was ophthalmology (50 cases, 77.0 %). 43 cases had their first ON attack and 22 cases had recurrent ON. Plasmapheresis (PP) was combined in 21 cases. The efficacy endpoint, changes in logarithm of the minimum angle of resolution (logMAR) after IVIG compared with preIVIG, showed statistically significant improvement in the AQP4-ON group at one week, 4 weeks, and 12 weeks after IVIG (p=0.015, p<0.001, p<0.001, respectively). In the MOG-ON group, excluding cases with combined PP, logMAR post IVIG did not improve significantly compared with preIVIG. Among the ION group, compared with preIVIG, logMAR at 4weeks and 12 weeks post IVIG were statistically significant improved (p=0.019, p=0.023, respectively). Adverse events occurred in 7 patients with IVIG. 4 of the 7 patients continued the IVIG treatment, and 3 patients discontinued it within 5 days.

**Conclusion:**

This study demonstrates that IVIG may be an effective new option for acute treatment of steroid-resistant ON as an add-on to conventional therapy.

**Supplementary Information:**

The online version contains supplementary material available at 10.1007/s10384-025-01210-6.

## Introduction

Optic neuritis (ON) is an acute disease in which visual acuity (VA) and visual field defects worsen within a few days and may cause irreversible damage in the chronic phase; hence, prompt treatment is crucial. Results of multicenter trials and epidemiological surveys in the United States [1] and Japan [2] show that steroid pulse (SP) therapy is the first choice for shortening the visual improvement duration; however, the long-term effectiveness of SP is unclear. Idiopathic ON (ION) is consistent with these survey results. Recently, autoimmune ON, such as anti-aquaporin-4 antibody (AQP4-Ab) and anti-myelin oligodendrocyte glycoprotein antibody (MOG-Ab) positive ON, has been identified as having clinically different characteristics from ION [3]. AQP4-Ab-positive ON is classified as neuromyelitis optica spectrum disorder (NMOSD) [4], a condition distinct from multiple sclerosis (MS), whereas MOG-Ab-positive ON is classified as MOG-IgG associated disorder (MOGAD) [5]. Both NMOSD and MODAD are central nervous system inflammatory demyelinating diseases that can present with severe ON and myelitis.

A recent nationwide survey of ON in Japan shows that patients with AQP4-Ab-positive ON were more likely to have poor VA after acute treatment than those with MOG-Ab-positive and double negative ON (AQP4-Ab-negative and MOG-Ab-negative) [6]. For autoimmune ON, especially AQP4-Ab-positive ON, it is recommended to initiate SP as early as possible after onset [7]. However, problems exist with steroid treatment resistance, poor visual improvement, and frequent recurrence [8]. Plasmapheresis (PP) has been used for severe ON that does not respond to acute steroid treatment, particularly AQP4-Ab-positive ON. Although PP improves the visual functions of ON [9–11], it is physically burdensome to patients and requires systemic management. Therefore, it is difficult for ophthalmologists to perform this treatment.

Recently, intravenous immunoglobulin (IVIG) has been reported as a new treatment option for steroid-resistant ON [12, 13]. IVIG involves less physical burden than PP, does not require complicated equipment or management by dialysis specialists, and may be administered by ophthalmologists alone. In Japan, a clinical trial on the efficacy of IVIG for ON suggests that it may be effective, especially for AQP4-Ab-positive ON, although there was no significant difference. However, in AQP4-Ab-negative cases, the visual improvement effect in the IVIG group was less than in the SP group [13]. The efficacy of IVIG for all ON, including ION and ON associated with MS has not been established [14, 15]. Reports on the indications and efficacy of IVIG for ON are limited. This study aimed to investigate the status of IVIG as an acute treatment for ON at multiple institutions in Japan and report the efficacy and safety of IVIG in a large number of cases.

## Materials and methods

### Participants

This study was a multicenter retrospective case series involving 30 facilities in Japan. To select the facilities, we first asked ophthalmologists or neurologists at major core hospitals in Japan, mainly university hospitals with extensive experience in treating ON and neuromyelitis optica, or facilities with council members of the Japanese Neuro-Ophthalmology Society (JANOS), to conduct a primary survey of cases of IVIG for ON. Among the facilities requested to complete the primary survey, we further requested a detailed secondary survey on the clinical characteristics of IVIG cases, particularly the results of ophthalmologic examinations such as VA after IVIG, and selected the 30 facilities that provided valid responses as research collaborating institutions. The facilities that did not respond and that administered IVIG but did not provide follow-up ophthalmologic examinations such as VA were excluded. The 30 facilities are located in each of the major regions, in 20 of the 47 prefectures of Japan.

Clinical data for relevant cases at each facility were obtained retrospectively from the medical records, and the investigator at each facility entered the information into a questionnaire. Clinical characteristics and laboratory test results of the subjects were collected using only existing materials. This study was conducted in compliance with the Declaration of Helsinki and was approved by the Ethics Committees of each participating institution (representative: Kitasato University Ethics Committee, approval no.: B22-115). Since this was a non-invasive observational study, it was ethically acceptable to obtain verbal informed consent from each patient after explaining the nature of the study.

Participants included those with ON in whom the VA did not improve sufficiently after acute treatment with SP, and IVIG was administered between January 1, 2020, and August 31, 2022. The criteria for steroid-resistant ON were cases in which the VA did not improve to 20/20 between the end of acute treatment with SP and the initiation of IVIG. The drug used for IVIG was freeze-dried sulfonated human immunoglobulin (Kenketsu Venilon^®^-I), at a dose of 400 mg (8 mL)/kg body weight/day by intravenous infusion. Patients in whom IVIG administration could be continued for 5 days were included. Regarding the combination of PP with SP and IVIG, the indications and timing of PP introduction were decided at the discretion of each facility.

The diagnostic criteria for ON were defined as follows, with reference to a previous report [3]. Clinical findings were defined as acute decreased VA or visual field defect, presence of a relative afferent pupillary defect (RAPD) in unilateral cases, reduced critical fusion frequency (CFF), and optional associated findings of orbital pain during eye movement and optic disc swelling. Magnetic resonance imaging (MRI) findings were defined as either contrast enhancement on gadolinium angiography or a high signal intensity on short tau inversion recovery (STIR) images in the optic nerve on the side associated with the above clinical findings, during the acute onset course. The site of the optic nerve lesion on MRI was defined as follows, based on a previous report [6]. The area toward the eyeball from half of the total length of the optic nerve in the orbit was classified as anterior, and the area toward the optic chiasm was classified as posterior. The same criteria were used for the MRI diagnosis of recurrent ON, including cases in which gadolinium-enhanced MRI could not be performed.

The exclusion criteria were cases in which ophthalmologic examinations including VA were not performed before and after IVIG administration, cases in which the above clinical and MRI findings of ON were not met, ON cases in which VA improved to 20/20 or better at the time of completion of SP and initiation of IVIG, and patients who discontinued IVIG administration due to adverse events within 5 days after initiating IVIG.

### Clinical characteristics

The clinical characteristics of patients with ON treated with IVIG include age, sex, ON types classified according to the status of autoantibodies (AQP4-Ab and MOG-Ab), complications with myelitis, main clinical department that administered IVIG (ophthalmology, neurology, others), patterns of visual field defects at onset of ON, presence or absence of optic disc swelling, and MRI findings—site of optic nerve lesion on MRI, and number of ON attacks at the time of IVIG administration. Combination treatment with IVIG was investigated, including the total number of SP treatments and days from onset of ON to SP and IVIG initiation. The onset of ON was defined as the onset of subjective symptoms. The above characteristics were investigated separately for all ON cases treated with IVIG, those treated by IVIG with concomitant PP, and those without PP. Additionally, for cases treated by IVIG with PP, total number of PP, and days from onset of ON to PP initiation were investigated.

In the evaluation of autoantibodies, AQP4-Ab was defined as AQP4-Ab-positive ON (AQP4-ON) when either the enzyme-linked immunosorbent assay (ELISA) or cell-based assay (CBA) was positive. In all cases in this study AQP4-Ab was measured either by ELISA or the CBA method. In Japan, only the ELISA method, which has lower sensitivity and specificity than the CBA method [16], is covered by health insurance. CBA is not available in some facilities; therefore, autoantibody test results were adopted by whichever of the two measurement methods had been used.

For MOG-Ab, we considered that some facilities were unable to use the CBA method, the only method available but is not covered by health insurance. Based on previously reported diagnostic criteria [5], MOG-Ab positive ON (MOG-ON) was defined only in cases in which antibodies were measurable and clearly positive. When clear positivity was not confirmed by MOG-Ab tests by CBA, the diagnostic criteria for MOG-ON were defined as cases with any of the following supporting clinical or MRI findings of the optic nerve: bilateral involvement, lesions extending vertically from the anterior part of the optic nerve, lesions extending to more than 50% of the total length of the optic nerve, optic perineuritis involving the optic nerve sheath, and severe optic disc edema accompanied by hemorrhage. Cases in which MOG-Ab could not be measured and did not meet the above diagnostic criteria for MOG-ON were excluded from the study.

ION was defined as a case in which AQP4-Ab and MOG-Ab positivity was not confirmed, MRI findings were similar to the characteristics of previously reported MS-associated ON [5], the lesions extended over a short area of the optic nerve and did not extend to the optic nerve sheath, and the case did not meet the above diagnostic criteria for MOG-ON.

Based on the above definitions, all ON cases treated by IVIG as well as cases treated by IVIG without PP, were classified into three groups: AQP4-ON, MOG-ON, and ION. The following items were compared among the three groups: age, sex, number of ON attacks, number of SP, days from the onset of ON to SP and IVIG initiation.

### Efficacy endpoints

Primary endpoint for IVIG efficacy for ON was the change in VA (logarithm of the minimum angle of resolution: logMAR) before and after IVIG, compared among the three groups of AQP4-ON, MOG-ON, and ION. For cases of ON in both eyes, VA values for both eyes were used. For cases in which VA values could be measured at each time point, the results were collected for cases in which they could be measured at the time of ON onset (onset), immediately before IVIG administration (preIVIG, after the end of acute SP), 1 week after IVIG administration (1w), 4 weeks after IVIG administration (4w), and 12 weeks after IVIG administration (12w). Changes in VA before IVIG were compared with preIVIG, defining onset as baseline, and changes in VA after IVIG were compared with 1w, 4w, and 12w defining preIVIG as baseline. Furthermore, as a subcategory analysis, excluding cases in which IVIG was used in combination with PP, the changes in VA over time were statistically compared similarly in the three groups: AQP4-ON, MOG-ON, and ION.

### Statistical analyses

Statistical analyses were performed using the statistical program language R (R version 4.1.1; R Foundation for Statistical Computing). For multiple comparisons of clinical characteristics among the three groups (AQP4-ON, MOG-ON, and ION), the Steel–Dwass test was used for continuous variables and Fisher’s exact test for nominal variables, depending on the distribution of each group. For the Steel–Dwass test, p<0.05 was considered statistically significant, and for the comparison of the three groups by the Fisher exact test, p<0.016 (0.05/3) was considered significant.

For statistical analysis, VA was presented in logMAR units, where counting fingers (CF) was converted to logMAR 2.6, hand movement (HM) to logMAR 2.9, light perception (LP) to logMAR 3.1, and no LP to logMAR 3.4 [17]. Changes in VA values over time at baseline and each time point were analyzed using linear mixed models, as some cases included data from both eyes. P-values were adjusted for multiple comparisons using Holm’s method [18]. P<0.05 was considered statistically significant for VA test results.

The above statistical analysis was performed for the all IVIG groups and the group excluding cases with concomitant PP, and the comparison was performed for the three groups: AQP4-ON, MOG-ON, and ION. Among the cases IVIG with PP, due to the small number of cases with MOG-ON and ION, statistical comparisons of clinical characteristics and the changes in VA were not performed among the three groups.

### Safety analysis

For safety evaluation of IVIG, the occurrence status and details of adverse events were investigated. The specific adverse events with relatively high frequencies were selected from the adverse events shown in the package inserts of the immunoglobulin, such as headache, decreased blood pressure, liver dysfunction, cytopenia, infection, anaphylaxis, and other adverse events were entered in the form of free description. Furthermore, information on whether to continue IVIG after any adverse events and measures taken during adverse events was obtained.

## Results

### Participant characteristics

Clinical characteristics of 65 patients (76 eyes) of ON treated with IVIG are shown in Table 1. In the classification of autoantibodies, AQP4-ON was the most common with 31 patients (47.7 %), MOG-ON with 13 patients (20.0 %), and ION with 21 patients (32.3 %). Of the four cases in which MOG-Ab was not measured, three cases were confirmed to be AQP4-Ab positive and were included in the AQP4-ON group. There were five pediatric patients aged <18 years included in the study, all in the MOG-ON group, and none in the AQP4-ON or ION groups. Five cases (2 men and 3 women) were complicated by myelitis, of which 3 were AQP4-Ab positive. Departments that administered IVIG were ophthalmology (50 patients, 77.0 %), neurology (11 patients, 16.9 %), and pediatrics (4 patients, 6.1 %). The most common visual field defect at onset was central scotoma (29 cases, 43.9 %), followed by total visual field defects (19 cases, 28.8 %), with a high proportion of cases with poor central vision. Optic disc swelling was observed in less than half of the patients (23 patients, 35.4%). MRI findings revealed inflammation in the posterior or entire length of the optic nerve in many cases, with a high proportion of retrobulbar ON. At the time of IVIG administration, 43 cases had their first ON attack, while 22 cases had two or more recurrences (maximum of six attacks). In past attacks, all patients had undergone SP treatment, and 7 patients had combined PP. Total number of SP was 2.2±1.0 (mean ± standard deviation [SD]) courses, with 53 cases with SP only before IVIG and 12 cases with SP before and after IVIG. The term from the onset of ON to SP initiation was 12.2±15.8 (1–101) days, whereas the term to IVIG initiation was 34.7±29.7 (4-142) days.

**Table 1:** Clinical characteristics of optic neuritis patients treated by intravenous immunoglobulin and classified according to with or without concomitant plasmapheresis

Characteristics	All ON cases with IVIG (n=65)	IVIG with PP (n=21)	IVIG without PP (n=44)
Age(years), mean ± SD (range)	51.7 ± 20.3 (5–87)	51.3 ± 17.6 (9–74)	51.9 ± 21.7 (5–-87)
Female, n (%)	46 (70.8)	19 (90.5)	27 (61.4)
ON type, n (%)			
AQP4-ON	31 (47.7)	16 (76.2)	15 (34.1)
MOG-ON	13 (20.0)	2 (9.5)	11 (25.0)
ION	21 (32.3)	3 (14.3)	18 (40.9)
Complicated with myelitis, n (%)	5 (7.6)	0 (0.0)	5 (11.4)
Main department, n (%)			
Ophthalmology	50 (77.0)	15 (71.4)	35 (79.6)
Neurology	11 (16.9)	5 (23.8)	6 (13.6)
Pediatrics	4 (6.1)	1 (4.8)	3 (6.8)
Visual field defect at onset, n (%)			
Central scotoma	29 (43.9)	9 (40.9)	20 (44.5)
Complete visual field loss	19 (28.8)	8 (36.4)	11 (25.0)
Optic disc swelling, n (%)	23 (35.4)	5 (23.8)	18 (40.1)
MRI findings: site of optic nerve lesion, n			
Anterior	13	6	7
Posterior	26	7	19
Entire length	19	5	14
Number of ON attacks, mean ± SD (range)	1.5 ± 1.1 (1–6)	1.7 ± 1.3 (1–6)	1.5 ± 0.9 (–5)
First ON attack, n (%)	43 (66.2)	13 (61.9)	30 (68.2)
Recurrent ON, n (%)	22 (33.8)	8 (38.1)	14 (31.8)
Number of SP, mean ± SD (range)	2.2 ± 1.0 (1-6)	2.4 ± 1.3 (1–6)	2.1 ± 0.8 (1–3)
Days from onset, mean ± SD (range)			
SP initiation	12.2 ± 15.8 (1–101)	9.9 ± 9.8 (1–37)	13.4 ± 18.2 (1–101)
VIG initiation	34.7 ± 29.7 (4–142)	42.2 ± 32.8 (8–-142)	31.1 ± 27.7 (4–142)

*ON* optic neuritis, *IVIG* Intravenous immunoglobulin, *PP* plasmapheresis, *SD* standard deviation, *AQP4-ON* anti-aquaporin-4 antibody positive optic neuritis, *MOG-ON* anti-myelin oligodendrocyte glycoprotein antibody positive optic neuritis, *ION* idiopathic optic neuritis, *MRI* magnetic resonance imaging, *SP* steroid pulse.

The clinical characteristics according to with or without of PP are shown in Table 1. PP was administered in combination with IVIG in 21 of the 65 patients (32.3 %), all of whom received it as an add-on therapy after SP. Number of PP was 4.8±3.0 (1–12) sessions, and the term from ON onset to PP initiation was 41.6±42.4 (5–206) days. Among the 21 patients with PP, 11 received PP followed by IVIG, and 10 received IVIG followed by PP. In the classification of autoantibodies, PP was most commonly combined with AQP4-ON in 16 of 31 patients (48.5 %), followed by 2 of 13 patients in MOG-ON and 3 of 21 patients in ION.

The clinical characteristics of all 65 cases of ON with IVIG were compared: 31 cases of AQP4-ON, 13 cases of MOG-ON, and 21 cases of ION (Table 2). In the statistical comparison of clinical characteristics among the three groups, significant differences were found between AQP4-ON vs MOG-ON (p=0.0002), and MOG-ON vs ION (p=0.0384) regarding age, with MOG-ON exhibiting a younger onset. No significant difference was observed in sex among the three groups; however, AQP4-ON tended to have a higher number of women. There were no significant differences among the three groups in the total number of SPs performed or the number of days from the onset of ON to the initiation of SP and IVIG. Among the 44 cases without PP, the clinical characteristics of 15 cases with AQP4-ON, 11 cases with MOG-ON, and 18 cases with ION were also similarly compared, and a statistically significant difference was found only in age between AQP4-ON and MOG-ON (p=0.0019) (Table 2).

**Table 2:** Comparison of clinical characteristics of all optic neuritis cases with intravenous immunoglobulin and cases without concomitant plasmapheresis among 3 groups (anti-aquaporin-4 antibody positive, anti-myelin oligodendrocyte glycoprotein antibody positive and idiopathic optic neuritis)

Characteristic	AQP4-ON	MOG-ON	ION	P-value			
All ON cases with IVIG	n=31	n=13	n=21	AQP4-ON vs MOG-ON	MOG-ON vs ION	AQP4-ON vs ION	Statistical Method
Age(years), mean ± SD	61.3±12.2	29.5±21.7	51.3±18.8	0.0002***	0.0384*	**0.0979**	*Steel-Dwass test*
Female, n (%)	26(83.9)	6(46.2)	14(66.7)	**0.0226**	**0.2962**	**0.1884**	*Fisher exact test*
Number of ON attacks, mean ± SD	1.5±1.0	2.2±1.4	1.3±0.7	**0.1080**	**0.0638**	**0.8739**	*Steel-Dwass test*
Number of SP, mean ± SD	2.0±1.2	2.1±1.0	2.5±0.6	**0.8711**	**0.7389**	**0.1134**	*Steel-Dwass test*
Days from onset,							
SP start, mean ± SD	14.2±20.7	6.4±4.1	12.6±10.2	**0.5553**	**0.1351**	**0.6373**	*Steel-Dwass test*
IVIG start, mean ± SD	33.5±26.4	34.8±34.7	36.5±32.5	**0.9926**	**0.9640**	**0.9173**	*Steel-Dwass test*
IVIG without PP	n=15	n=11	n=18				
Age(years), mean ± SD	65.1±11.7	32.2±22.5	52.9±19.3	0.0019**	**0.0917**	**0.1388**	*Steel-Dwass test*
Female, n (%)	12(80.0)	4(36.4)	11(61.1)	**0.0641**	**0.3622**	**0.4264**	*Fisher exact test*
Number of ON attacks, mean ± SD	1.2±0.6	2.1±1.4	1.4±0.8	**0.0526**	**0.1457**	**0.7829**	*Steel-Dwass test*
Number of SP, mean ± SD	1.8±0.9	2.1±1.0	2.4±0.5	**0.6555**	**0.8491**	**0.0799**	*Steel-Dwass test*
Days from onset,							
SP start, mean ± SD	19.7±28.0	6.4±4.2	12.2±10.0	**0.2354**	**0.1708**	**0.9999**	*Steel-Dwass test*
IVIG start, mean ± SD	29.8±26.8	25.3±13.8	35.9±34.8	**0.9867**	**0.8956**	**0.8072**	*Steel-Dwass test*

*For the Steel-Dwass test, p<0.05 was considered statistically significant, and for the comparison of three groups by the Fisher exact test, p<0.016(0.05/3) was considered significant.*

*ON* optic neuritis, *IVIG* intravenous immunoglobulin, *AQP4-ON* anti-aquaporin-4 antibody positive optic neuritis, *MOG-ON* anti-myelin oligodendrocyte glycoprotein antibody positive optic neuritis, *ION* idiopathic optic neuritis, *SD* standard deviation, *SP* steroid pulse, *PP* plasmapheresis.

### Efficacy endpoints

**Fig. 1:**
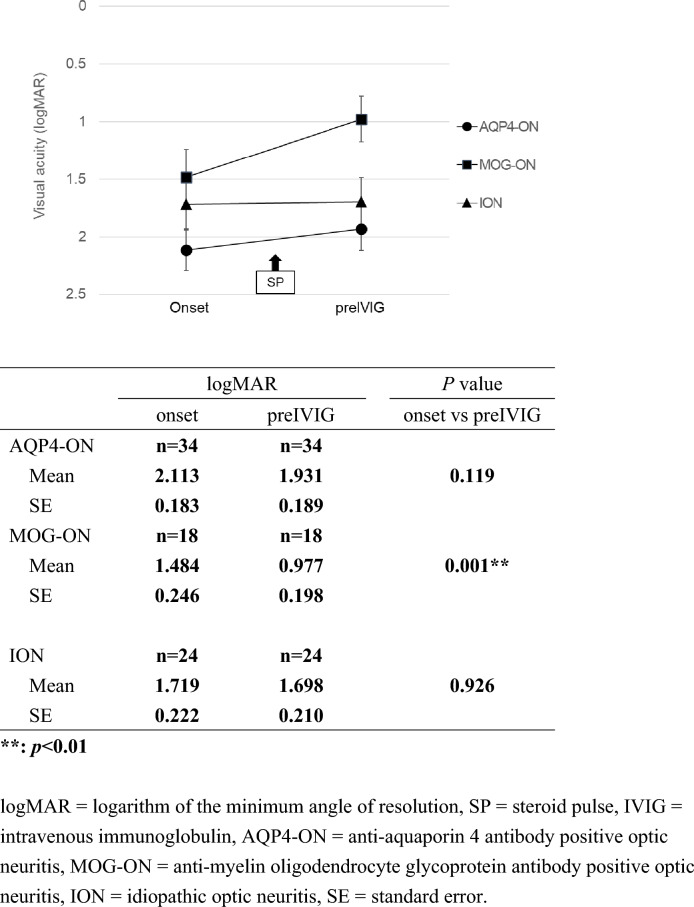
Changes in logMAR (mean ± standard error) before intravenous immunoglobulin among three optic neuritis groups.**: p<0.01, *logMAR* logarithm of the minimum angle of resolution, *SP* steroid pulse, *IVIG* intravenous immunoglobulin, *AQP4-ON* anti-aquaporin 4 antibody positive optic neuritis, *MOG-ON* anti-myelin oligodendrocyte glycoprotein antibody positive optic neuritis, *ION* idiopathic optic neuritis, *SE* standard error.

**Fig. 2:**
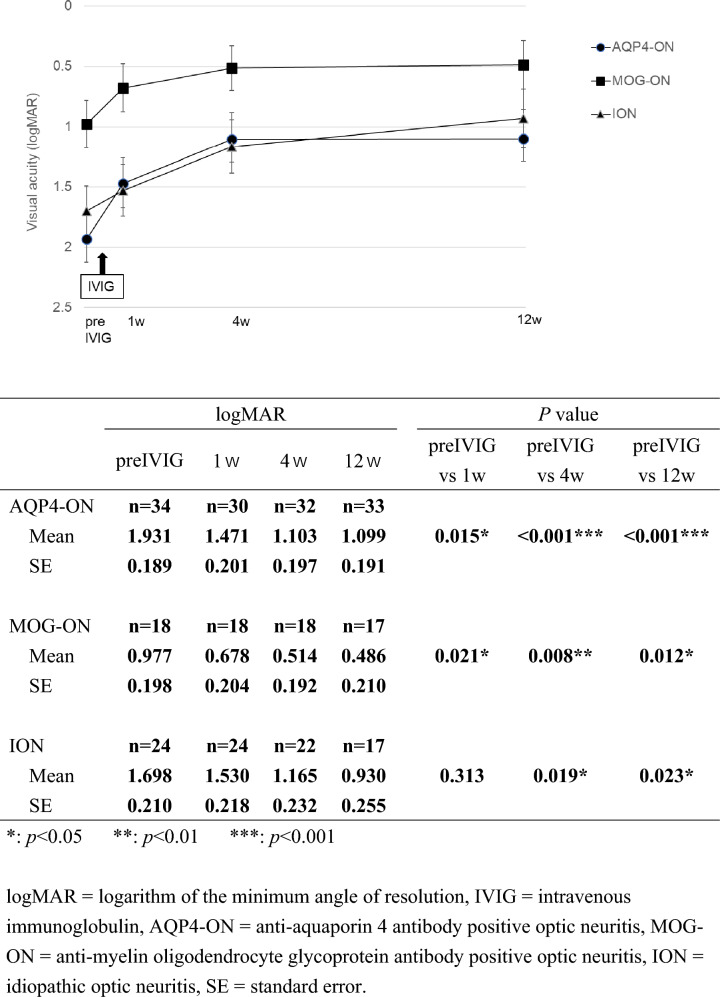
Changes in logMAR (mean ± standard error) after intravenous immunoglobulin among three optic neuritis groups. *: *p*<0.05 **:* p*<0.01 ***:* p*<0.001. *logMAR* logarithm of the minimum angle of resolution, *IVIG* intravenous immunoglobulin, *AQP4-ON* anti-aquaporin 4 antibody positive optic neuritis, *MOG-ON* anti-myelin oligodendrocyte glycoprotein antibody positive optic neuritis, *ION* idiopathic optic neuritis, *SE* standard error

In cases of bilateral ON, VA of both eyes was evaluated; 34 eyes (unilateral 28 eyes and bilateral 3cases, 6 eyes) with AQP4-ON, 18 eyes (unilateral 8 eyes and bilateral 5cases, 10 eyes) with MOG-ON, and 24 eyes (unilateral 18 eyes and bilateral 3cases, 6 eyes) with ION were examined. Figure 1 shows the change in VA before IVIG, and Figure 2 shows the change in VA after IVIG.

Among the three groups, AQP4-ON had the worst VA before IVIG at both onset and preIVIG, whereas MOG-ON had a better VA than AQP4-ON and ION. In MOG-ON group, a significant improvement was observed with logMAR of 0.977 ± 0.198 (mean ± standard error [SE]) at preIVIG compared with logMAR of 1.484 ± 0.246 at onset (p = 0.001). In contrast, no significant change in VA was observed in the AQP4-ON and ION groups when comparing onset and preIVIG (Fig. 1).

In VA after IVIG, in AQP4-ON group, significant improvement was observed at all time points, 1w (logMAR: 1.471±0.201, p=0.015), 4w (logMAR: 1.103±0.197, p<0.001), and 12w (logMAR: 1.099±0.191, p<0.001), after IVIG, compared with preIVIG (logMAR: 1.931±0.189). In the MOG-ON group, compared with preIVIG, significant improvements were observed at 1w (p=0.021), 4w (p=0.008), and 12w (p=0.012) after IVIG. In the ION group, no significant improvement was observed at 1w after IVIG, but significant improvement was observed at 4w (p=0.019) and 12w (p=0.023) compared with preIVIG. (Fig. 2).

**Fig. 3:**
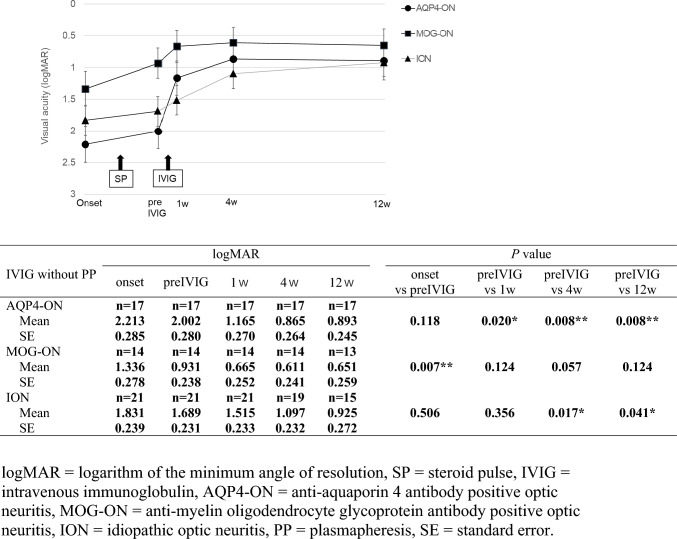
Changes in logMAR (mean ± standard error) before and after intravenous immunoglobulin without plasmapheresis among three optic neuritis groups. *logMAR* logarithm of the minimum angle of resolution, *SP* steroid pulse, *IVIG* intravenous immunoglobulin, *AQP4-ON* anti-aquaporin 4 antibody positive optic neuritis, *MOG-ON* anti-myelin oligodendrocyte glycoprotein antibody positive optic neuritis, *ION* idiopathic optic neuritis, *PP* plasmapheresis, *SE* standard error

In 52 eyes of 44 cases without PP, 17 eyes of 15 cases had AQP4-ON, 14 eyes of 11 cases had MOG-ON, and 21 eyes of 18 cases had ION (Fig. 3). Compared between onset and preIVIG, only the MOG-ON group showed significant improvement in VA (p=0.007). Regarding the change from preIVIG to after IVIG, VA improved significantly at 1w (p=0.020), 4w (p=0.008), and 12w (p=0.008) in the AQP4-ON group and at 4w (p=0.017) and 12w (p=0.041) in the ION group, whereas no significant change was observed at 1w, 4w, or 12w in the MOG-ON group.

As supplement, in 17 eyes of 16 cases with PP in the AQP4-ON group, (A) PP had been performed before IVIG in 10 eyes of 9 cases (1 bilateral case), and (B) PP was performed after IVIG in 7 eyes of 7 cases. VA tended to be worse in group (A) at all times: onset, preIVIG, and 1w, 4w, and 12w after IVIG, but no statistically significant difference was observed when compared with group (B) (Supplement).

### Safety analysis

Adverse events were observed in seven of 65 patients (10.8 %) after IVIG initiation for ON. Of the 7 patients with adverse events, 4 patients continued IVIG administration and 3 patients discontinued. Adverse events in the 4 patients who continued IVIG included skin itching, chest discomfort, liver dysfunction, and infection, which improved with observation and conservative treatment (Table 3). The adverse events, case details, and outcomes of the 3 patients in which IVIG was discontinued administration are shown in Table 4. Case 1, who presented with cytopenia, was a 70-year-old woman with AQP4-ON and a recurrent case (second attack). logMAR was 2.000 at onset and improved to 0.222 after 3e courses of SP. PP was added after IVIG was discontinued, but final logMAR remained at 0.699. Case 2, who presented with stroke with no symptoms, was a 77-year-old woman with AQP4-ON and a first-onset case. logMAR was 0.523 at onset and remained unchanged after one course of SP. After IVIG was discontinued, logMAR improved to 0.097 without additional treatment. Case 3, who presented with headache, nausea, and arm numbness, was a 20-year-old man with ION and a recurrent case (second attack). logMAR was 0.699 at onset and remained unchanged after one course of SP. After IVIG was discontinued, logMAR remained at 1.000 without additional treatment.

**Table3:** Summary of cases of optic neuritis in which intravenous immunoglobulin was continued after adverse events

Case	1	2	3	4
Age (years)	9	25	21	36
Sex	female	female	male	female
ON type	MOG-ON	ION	ION	AQP4-ON
ON attacks	1	1	1	1
Number of SP	3	2	3	3
Add on therapy	PP	PP	None	None
Adverse events	Skin itching	Chest discomfort	Liver dysfunction	Infection
Deal with Adverse events	Oral anti-allergy medication	Slow down intravenous administration	Observation	Observation

*ON* optic neuritis, *AQP4-ON* anti-aquaporin-4 antibody positive optic neuritis, *MOG-ON* anti-myelin oligodendrocyte glycoprotein antibody positive optic neuritis, *ION* idiopathic optic neuritis, *SP* steroid pulse, *PP* plasmapheresis.

**Table 4:** Summary of cases of optic neuritis in which intravenous immunoglobulin was discontinued after adverse events

Case	1	2	3
Age(years)	70	77	20
Sex	female	female	male
ON type	AQP4-ON	AQP4-ON	ION
ON attacks	2	1	2
Number of SP	3	1	1
Add on therapy	PP	None	None
Visual acuity (logMAR)			
Onset	2.000	0.523	0.699
preIVIG (afterSP)	0.222	0.523	0.699
After treatment	0.699	0.097	1.000
Adverse events	Cytopenia	Stroke (no symptoms)	Headache, nausea, and arm numbness

*ON* optic neuritis, *AQP4-ON* anti-aquaporin-4 antibody positive optic neuritis, *MOG-ON* anti-myelin oligodendrocyte glycoprotein antibody positive optic neuritis, *ION* idiopathic optic neuritis, *SP* steroid pulse, *PP* plasmapheresis, *logMAR* logarithm of the minimum angle of resolution.

## Discussion

We investigated IVIG efficacy for ON in Japan and obtained results for several cases. Clinical characteristics of ON cases treated with IVIG suggest that IVIG may have been necessary for cases in which visual function had not improved sufficiently and visual impairment was present at the time of onset due to central scotoma and total visual field defects. The average number of days from ON onset to SP and IVIG initiation was within 2 weeks and >30 days, respectively. In this study, the ON onset was defined as the time when symptoms first appeared, and it often took several days from the onset of symptoms until ON was diagnosed at specialized facilities, which resulted in a number of days longer than that stated above. In addition, IVIG is approved as an additional treatment after initial SP treatment immediately after ON onset, and in this study, SP was performed for an average of > two courses, and IVIG was administered after the effectiveness of SP was evaluated. In the classification of ON types based on autoantibodies, we found that IVIG was administered not only for AQP4-ON, but also for MOG-ON and ION, for which SP was ineffective. The clinical characteristics of cases treated with IVIG showed similar trends to those reported in a previous report [6], in that AQP4-ON was more frequently identified in women and MOG-ON was younger at onset and had a higher frequency of attacks.

### Efficacy of IVIG for AQP4-Ab-positive ON

Before it was approved for ON, IVIG had been administered as a treatment for various diseases, such as myasthenia gravis, blood and skin diseases, and neuropathy. The mechanism of IVIG is speculated to be involved in the pathology of autoimmune diseases. Although details of that mechanism are unclear, various factors, such as actions via the immunoglobulin variable region—F(ab')_2_—and constant region—Fc receptors—are thought to be involved [19, 20]. In animal models, two mechanisms, complement-dependent cytotoxicity and antibody-dependent cellular cytotoxicity, are involved in the pathology of AQP4-Ab-positive NMOSD. Immunoglobulin G is involved in these pathways and acts antagonistically against AQP4-Ab, thereby reducing NMO-IgG [21].

IVIG is considered applicable for the acute treatment of AQP4-ON from pathological and mechanism of action viewpoints, and IVIG was effective in AQP4-Ab positive ON and NMOSD [12, 13, 22, 23]. Nakao et al. report that VA improved with IVIG in three of four cases of AQP4-Ab positive ON [12]. In this report, all three cases of improvement were confirmed by MRI before IVIG administration; cases in which improvements did not occur were considered ineffective because IVIG had already begun to atrophy the entire optic nerve. This report suggests that IVIG alone is insufficient to improve VA, and that the effectiveness may be achieved by the strong anti-inflammatory effect of SP and the regulatory effect of IVIG on humoral immunity. Factors that suggest IVIG may be more effective include a short time between ON onset and treatment initiation, AQP4-Ab positivity, and enhancement (presence of inflammation) of the optic nerve on contrast MRI [12].

Mimura et al. conducted a multicenter, double-blind, randomized, parallel-group controlled study in Japan and report the effectiveness of IVIG compared with SP therapy for acute refractory ON [13]. In this study, the changes in visual acuity (logMAR) 2 weeks after treatment initiation from before treatment initiation was not superior to that in the SP group in an analysis of variance, with AQP4-Ab positivity and negativity as factors. Contrastingly, the number of participants whose logMAR VA improved by ≥ 0.3 two weeks after treatment initiation was higher in the IVIG group than that in SP group. Moreover, visual prognosis was significantly better in AQP4-Ab positive cases than that in control SP group, but was poor in negative cases. Furthermore, in IVIG group, even when central visual field did not improve, peripheral visual field significantly improved compared with that in SP group.

In our study, the addition of IVIG after SP significantly improved VA in AQP4-ON: VA improved significantly after 1 week of IVIG, with further improvements after 4 and 12 weeks, and VA remained unchanged after 4 and 12 weeks of IVIG. Therefore, the effect of IVIG was more pronounced after a certain amount of time had passed since administration, and VA, once improved by IVIG, was maintained without decline. A previous report [13] examined visual function 2 weeks after IVIG; however, the results of this study suggest that IVIG may maintain its effectiveness in improving VA for an even longer period.

On the other hand, in our study, the superiority of IVIG over PP and the indications for the combination of IVIG and PP are debatable. Even in groups excluding those in which PP was combined, VA improved significantly at 1, 4, and 12 weeks after IVIG, indicating that the addition of IVIG alone without PP after SP in AQP4-ON may be effective in improving visual function. However, regarding the timing of PP, there were a similar number of cases in the PP combination group who received PP before and after IVIG, but the number of cases in each group was limited, so a statistical difference between the two could not be proven, and it was also difficult to directly compare VA improvement effects between the groups with and without PP. In particular, it is recommended that PP be prioritized over IVIG for severe SP-resistant AQP4-ON, as visual impairment may become irreversible over time from onset. In this study, the mean number of days from the onset of ON to the initiation of both IVIG and PP was more than one month, with a large difference ranging from several days to several weeks depending on the case. Further studies are needed to examine whether a shorter introduction of IVIG is associated with a better prognosis for VA improvement and the indications for additional treatment with IVIG or PP in severe cases.

### Efficacy of IVIG for MOG-Ab-positive ON and ION

For AQP4-Ab-negative ON, IVIG is appropriate for cases where autoimmune pathology is suspected and other treatments, such as SP therapy, have minimal or no effect on visual function improvement, or other treatments are difficult to use. However, its efficacy has not been established for MOG-ON, ION, and ON associated with MS other than AQP4-ON. Previous reports show that IVIG group significantly improved in vision compared with that of control group for ON associated with MS [24], whereas others indicate that the mechanism of action underlying the efficacy of IVIG is unclear because the pathology of MS is different from that of NMOSD [19].

Mizui et al. report the efficacy of IVIG for ON, including cases other than AQP4-Ab-positive cases [25]. IVIG was administered to six cases (seven eyes), with two cases each of AQP4-ON, MOG-ON, and ION. Best-corrected VA was evaluated 1 week to 1 month after IVIG during a mean observation period of 16.7 (range 6–48) months. Five of the seven eyes showed improvement to final decimal VA of ≥1.0 after IVIG, indicating that there were effective cases for each type of ON. In this report, in two cases–one each of AQP4-ON and MOG-ON–whose VA did not improve with IVIG, the final VA improved with the addition of PP after IVIG.

In this study, the MOG-ON group showed significant VA improvement from the onset of symptoms after SP therapy before IVIG, and the characteristics of the MOG-ON patients responding relatively well to steroids were consistent with previous reports. Furthermore, a significant improvement in VA was observed 1, 4 and 12 weeks after IVIG compared with before IVIG, indicating that further improvement in visual function can be obtained with IVIG. The improvement in VA after IVIG may have been due to the effect of the preceding SP manifesting itself over time or the addition of IVIG to SP resulting in a synergistic effect. However, in this study, no significant change in VA after IVIG was observed in the group excluding cases with concomitant PP, so it was difficult to evaluate the efficacy of adding IVIG alone to SP in the MOG-ON group. To examine the pure efficacy of IVIG in the MOG-ON group, it was considered necessary to compare changes in VA between groups in which SP and IVIG were administered within the same number of days, as well as to compare changes in VA between groups in which only SP and only IVIG was administered from the onset. Although MOG-ON often responds well to SP treatment and only a small number of cases have visual function so poor that PP is required, the addition of IVIG may be an effective option, especially when VA does not improve sufficiently to 20/20 even after one or two courses of SP. In addition, if IVIG can reduce the total amount of steroids used, including during the acute phase, it may have the benefit of reducing serious side effects caused by steroids.

In the ION group, VA improved significantly 4 and 12 weeks after IVIG in both the overall group including cases with concomitant PP, and the SP+IVIG group without PP. Although only a small number of patients with poor visual function required the concomitant PP and it was shown that IVIG in combination with SP may tend to improve VA to some extent, it is unclear whether the efficacy is related to a mechanism of action similar to that of AQP4-ON. ION was diagnosed by exclusion when AQP-Ab and MOG-Ab were negative. It was often difficult to understand the pathology, predict visual function prognosis, and response to treatment of ION. The efficacy of IVIG in ION and ON associated with MS, which have different clinical characteristics from AQP4-ON and MOG-ON, may require investigation into more precisely defined case groups.

### Safety of IVIG for ON

Adverse events of IVIG occur in less than 10% of patients with neurological disorders [19]. Adverse events may occur during or immediately after IVIG administration or slightly later after administration. The most common adverse event occurring during or immediately after administration are mild-to-moderate headaches. In most cases, conservative follow-up is possible, and prognosis is good. Serious adverse events caused by IVIG are rare; however, liver dysfunction, aseptic meningitis, decreased renal function, thrombocytopenia, and thromboembolism are reported [26–28]. Moreover, because immunoglobulin preparations are refined from human blood, there is a risk of infection transmission, similar to blood transfusions. Therefore, intravenous injections are recommended to be administered slowly for 30 min on the first day, as rapid injection speed may cause a drop in blood pressure or anaphylaxis, and the administration speed should be gradually increased after confirming that no adverse events have occurred.

In this study, seven of 65 patients, a frequency comparable to that reported in previous studies [19], experienced adverse events after IVIG administration. Four Patients who continued IVIG after the occurrence of adverse events were judged to have mild symptoms that improved with follow-up observations and conservative treatment. Among the 3 patients who discontinued administration, those with headache, nausea, and numbness in the arms were young, but the patients with cytopenia and stroke without symptoms were elderly (> 70 years old), which may have influenced the decision to discontinue administration. No cases of blood pressure drop or serious infection were observed immediately after IVIG administration. The frequency of adverse events after IVIG is not high, and there is no consensus on how to deal with adverse events or when to discontinue administration. It may be desirable to consider each case based on age and the presence of underlying diseases.

### Study limitations

This was a retrospective observational study using existing samples and the effectiveness of the IVIG group was not compared with that of a control group. At the stage of selecting the facilities to be included in this study, the proportion of ophthalmologists was high and of neurologists low, so we were unable to comprehensively cover cases of IVIG treatment for ON treated by neurologists, and it is possible that there are more cases with different characteristics. The number of days from onset at which the effect of SP before IVIG was deemed ineffective varied from case to case, and in some cases it was difficult to determine whether the VA improvement was due to IVIG or the natural history. In addition, this study did not compare the effectiveness of IVIG and PP monotherapy, and it is unclear whether IVIG is more effective than PP, especially in cases of poor visual function, such as AQP4-Ab positive ON. To prove the efficacy of IVIG in the MOG-ON group, data on significant improvements in VA before and after IVIG without concomitant PP are required. For recurrent ON, the possibility cannot be ruled out that VA after treatment of the previous attacks may have influenced the evaluation of VA after IVIG, and although this was not done in this study, it would have been necessary to compare the groups with good and poor VA after treatment following previous attacks. Further investigation is required to determine the indications and factors that make IVIG effective in each case of positive or negative autoantibodies.

The results of this study indicate that IVIG may be an effective option for acute treatment of ON, and can be added to conventional methods such as SP and PP. Our results show that in patients with AQP4-Ab-positive ON, VA improved one week after IVIG without concomitant PP, and that improvement was maintained over the long term, even after 12 weeks. IVIG may be suitable for cases in which SP is ineffective or if further treatment is desired, and PP is difficult to perform. Future long-term observational studies involving a larger number of cases are needed to examine the superiority of IVIG over other treatments.

## Supplementary Information

Below is the link to the electronic supplementary material.

Supplementary file1 (DOCX 32 KB)
