# Supplementary material for: Intravenous immunoglobulin for the acute treatment of refractory optic neuritis in Japan
Source: Jpn J Ophthalmol. 2025 May 16;69(5):775–88. doi: 10.1007/s10384-025-01210-6 (PMC12391192; doi:10.1007/s10384-025-01210-6)
Supplement: Supplementary file 1 — Supplementary file1 (DOCX 32 KB) [file 10384_2025_1210_MOESM1_ESM.docx]

**Supplement.** Changes in logMAR (mean ± standard error) before and after intravenous immunoglobulin with plasmapheresis of anti-aquaporin 4 antibody positive optic neuritis groups.

.


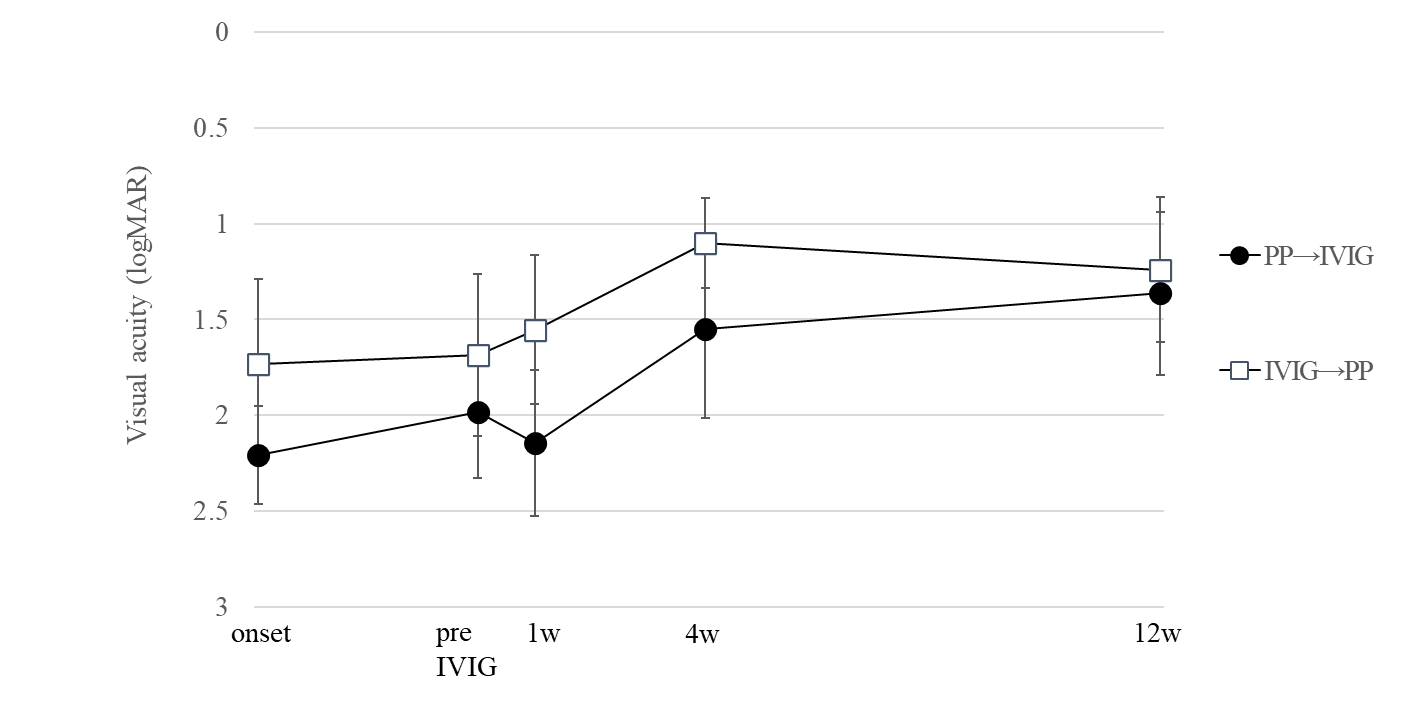


| AQP4-ON | logMAR | | | | |
| --- | --- | --- | --- | --- | --- |
| IVIG with PP | onset | preIVIG | 1ｗ | 4ｗ | 12ｗ |
| PP→IVIG  Mean  SE | **n=10**  **2.210**  **0.256** | **n=10**  **1.982**  **0.346** | **n=7**  **2.146**  **0.382** | **n=9**  **1.552**  **0.461** | **n=10**  **1.363**  **0.426** |
| IVIG→PP  Mean  SE | **n=7**  **1.734**  **0.445** | **n=7**  **1.685**  **0.425** | **n=6**  **1.553**  **0.389** | **n=6**  **1.103**  **0.235** | **n=6**  **1.240**  **0.379** |

logMAR = logarithm of the minimum angle of resolution, IVIG = intravenous immunoglobulin, PP = plasmapheresis, AQP4-ON = anti-aquaporin 4 antibody positive optic neuritis, SE = standard error.
